# Book Reviews

**Published:** 1832-04

**Authors:** 


### BIBLIOGRAPHICAL NOTICES.

*Letters on the Cholera.* By WHITELAW AINSLIE, M.D., M.R.A.S., M.R.S.E.—Wilson, London.

*A Letter to the President of the Westminster Medical Society, on Cholera.* By J. WEBSTER, M.D., Physician to St. George's and St. James's Dispensary.

*The Laws and Progress of the Epidemic Cholera, illustrated by Facts and Observations.* By THOMAS HANCOCK, M.D.—Hamilton and Co., London.

*Substance of a Lecture on Cholera, read before a Meeting of the Medical Profession of Liverpool, on the* 18*th January,* 1832. By D. BAIRD, M.D., Physician to the Fever Hospital.—Hatchard and Son, London.

*Cholera, its Character and Treatment; with Remarks on the Identity of the Indian and English, and a particular Reference to the Disease as now existing at Newcastle and its Neighbourhood.* By CHARLES TURNER THACKRAH.—Longman and Co., London.

WE are desirous of submitting to our readers a concise outline of all that is said upon the subject of Cholera. To give more than a very concise outline of the various publications upon this theme, would be impracticable within the limits to which we must confine ourselves upon any one subject, how great soever may be its importance.

In 1825, Dr. AINSLIE published his "Observations on the Cholera Morbus, in a Letter addressed to the Honourable the Court of Directors of the East India Company," and the present "Letters" from the same author are written in pursuance of the theory he had adopted regarding cholera when he gave his former work to the public. Dr. Ainslie's "original idea, that the immediate, or rather the exciting, cause of the disease, is an *acescency of a peculiar kind*, is nothing changed." In adopting this notion, Dr. A. finds himself supported by the high authority of BOERHAAVE, who speaks of "the most terrible *choleras* and *convulsions* being produced by an *acid** acrimony in the first passages." When on the medical staff of Southern India, in the latter end of 1815, Dr. Ainslie was particularly struck with the then, to him, new appearance which cholera was assuming: in place of the tractable disorder he had ever found it up to that period, he not unfrequently observed it put on a much more alarming aspect, characterised by (in addition to violent vomiting and purging of a whitish-coloured fluid,) a great sinking of the nervous energy, excruciating spasms, fixed hollow eye, feeble voice, altered expression of countenance, and occasional asphyxia."

A severe, and indeed apparently hopeless, case is referred to, in which ordinary remedies were unavailing. It at length occurred to Dr. A. that "perhaps some irritating acrimony or other" required correcting; and he gave the patient a full dose of calcined magnesia, *not* in milk, but in a little tepid water: this medicine was retained, the patient soon fell asleep, and in the morning had

* BOERHAAVE's Practical Aphorisms, No. 63.

no complaint but weakness. From this time, both in India and England, Dr. A. has given in cholera, *in the first instance*, an *antacid*, and with continued advantage.

Various writers have recently spoken very highly of a similar mode of treatment. Dr. Paul Slade Knight, in the Medical Gazette for March 17th, recommends one-drachm doses of the carbonate of soda in cholera, as he had also been led to infer that a "peculiar morbific acid is the cause of all the frightful tortures and deaths." Mr. Goss, in his "Practical Remarks on the Disease called Cholera," assures us that the carbonates of soda and ammonia have been found of the greatest utility. At Sunderland, again, Mr. Torbock tried the carbonate of soda, with the most successful results. We could mention many other practitioners who speak very favorably of the mode of treatment suggested by Dr. Ainslie: some of them, indeed, differ from him as to the *views* with which they prescribe antacids, but all who have employed them speak favorably of their effects.

After giving this useful, because tangible and practical fact, Dr. Ainslie enters upon more debateable points: he endeavours to shew, and with much ingenuity, we grant, that the "peculiar and morbid acescency" depends upon some modification of atmospheric electricity.

Dr. Webster is not satisfied that the disease now prevailing is altogether an unknown one in this country: from all that is yet known of this disease, he thinks it would appear to be either a most aggravated form of pestilent or *typhous* fever, in which the cold stage is so overpowering as even to kill the individual at the very first attack; or "it may be called the English cholera, whose cold stage is much more sudden and fatal than is in most years, or in modern times, observed." The malady now existing in different parts of London "*may* be called" by whatever denomination each observer chooses to apply to it: for ourselves we must say, that the term "English cholera" ought not, and cannot with propriety be applied to those cases we have seen in Southwark and St. Giles's. Dr. Webster is a non-contagionist.

Dr. Hancock's "business is not with the medical, but the natural history" of the epidemic cholera: he is anxious to bring some of its phenomena within the established laws of other pestilential epidemics, and to shew that, notwithstanding its course is marked by many eccentricities, it is still subject, in its movements and the extent of its ravages, to certain physical laws, "which seem, much more than any casualties, to influence its progress and propagation." Dr. Hancock's work is well worth perusing, if it were only as an example of the impartial and dispassionate manner in which he considers the subject of contagion, which has so frequently, and especially by many of the orators in our Medical Societies and authors of letters in the newspapers, been conducted with the party-spirit of politicians, instead of the temperance of scientific men, whose sole object should be to elicit the truth. Dr. Hancock's opinions respecting contagion we believe to be perfectly correct, and we imagine that the number of those whom he would class under the title of "ultra-contagionists" is extremely limited. "According to my own modified, and I hope not incorrect, views of the doctrine of contagion, in which I regard it only as a subordinate power, acting chiefly on those predisposed, and not by any means the sole cause of epidemic pestilence, I see few difficulties, compared with those of the *ultra*-contagionists, if, without offence, I may so designate many candid and zealous medical observers."

Dr. Baird's "Lecture" contains an interesting account of his visit to Newcastle and its neighbourhood, while cholera was raging there, together with such facts and observations as he could collect relative to the nature and treatment of the disease. Dr. B. very truly observes, that "our attention has been too much diverted from the path of sober investigation by a rage for nostrums and specifics, all of which have failed, and in the ratio of their failure has the confidence of the public declined. We do not find that this lecture contains any particular novelty, but it is, notwithstanding, creditable to the author, from its presenting, in a condensed form, a clear account of the history of the disease, and the best modes of treatment that have been suggested to relieve it.

From Mr. Thackrah's pen the profession has at various times derived much information upon very interesting topics: we are happy, therefore, to find him claiming our attention upon the subject of Cholera. Mr. Thackrah first gives a good practical account of the chief forms under which cholera presents itself: 1, the mild autumnal bilious flux; 2, congestive or purple cholera; 3, cholera with marked defect of secretions. He then enters upon the description of "an English epidemic" which produced severe effects and considerable alarm in the autumn of 1825: he speaks of this disease as it occurred in the neighbourhood of Leeds; and, after having given an account of the epidemic, he refers to notes of cases which have occurred in different years under his own observation, or that of other practitioners in the neighbourhood. We have attentively perused the description Mr. Thackrah gives of this epidemic, and we cannot but yield our assent to the inference he draws from what *he* and others in his vicinity observed. In the cases he relates, the signs which are said to characterize the Indian cholera are certainly found in a marked and decided degree: but we must not allow our readers to suppose that Mr. T. infers more from his facts than they will fairly justify. He does not offer the cases he adduces as *fair* samples of English epidemics. "They are rare." "They shew not what English cholera generally is, but what it can be; the appalling form which it sometimes presents." When the disease does occur in this aggravated form, Mr. Thackrah maintains it does not differ from the malignant cholera, the spasmodic cholera of India; and the evidence he brings forward, in our opinion, fully justifies the conclusion. But we must observe, at the same time, that the epidemic of 1825 which Mr. Thackrah describes as having occurred at Leeds and in its vicinity, differs widely from the cholera which prevailed in London in the same year. In the latter place, the disease, as far as we know from our own observation, and the information of many other medical practitioners, made no such approach to the Indian cholera as that pointed out, we have no doubt very correctly, by Mr. Thackrah. It is true that many of the London patients exhibited great and even alarming symptoms of exhaustion, but that exhaustion always appeared to depend upon the excess and continuance of vomiting and purging; and, when the disease did prove fatal, it was only in such subjects as were previously so completely worn out by poverty and chronic ailments, as to be incapable of bearing any additional demand upon their feeble powers.

Mr. Thackrah gives a good sketch of the general principles by which our treatment of cholera should be guided, and offers many interesting remarks as to the effects of certain remedies. He considers "ammonia a particularly valuable remedy, the best of diffusible stimulants;" and is surprised to find that it has been so rarely noticed. The importance of promptly attending to the invasion of the disease, is properly insisted upon. Mr. T. believes that nine-tenths of the worst cases of cholera are preceded for a few days by disorder which would never have been succeeded by fatal disease, had it been early regarded.

In the course of the last month we have seen several cases of simple but excessive diarrhœa, in which symptoms of great exhaustion very rapidly supervened: they were quickly attended to, and were relieved in the course of a few hours; but we have no doubt that if appropriate remedies had not been speedily applied, the lives of these patients would have been placed in great jeopardy. In the cases we refer to, the Liq. Opii Sedat. was given with immediate advantages; mild purgatives were subsequently administered.

---

*A Series of Experiments, performed for the purpose of shewing that Arteries may be obliterated without Ligature, Compression, or the Knife.* By Benj. Phillips.—8vo. pp. 66. Longman, London.

Very extended opportunities of observing the progress and the effects of aneurism, and the occasional difficulties and dangers of the operation by ligature, induced Mr. Phillips "to ponder long and anxiously on the possible means of lessening the difficulties, and avoiding the dangers of that operation." He had long believed, that if he could devise any mode by which inflammation might be excited in the parietes of an artery, that inflammation would be succeeded by coagulation, and a consequent obliteration of the artery. The object of this pamphlet is to explain the circumstances which, occurring from time to time, tended to strengthen this conviction, and at the same time to point out the mode adopted for testing the correctness of the opinion by actual experiment, "and the complete and entire success of the experiments made." From his own observations, and the remarks of others upon the subject, Mr. Phillips feels justified in concluding that an artery may be obliterated,

"First, by the existence of inflammation within its coats, producing adhesion;

"Secondly, by lessening the force of the circulating fluid, without the existence of inflammation, by the formation of a clot:

"Thirdly, by the intermedium of both these causes in concert;

"Fourthly, that the existence of a foreign body in the canal, in the absence of other diseases, will arrest the progress of the circulating fluid, and favor the production of a coagulum; and,

"Fifthly, by the introduction of a foreign body into the canal, creating or accompanied by inflammation.

"To establish the correctness of the latter positions, I engaged in the series of experiments I am now about to detail. The animals upon which I operated were, with only two exceptions, dogs; the arteries upon which I experimented, the femorals and the carotids, which were selected simply from the greater facility with which they may be transfixed."

Ten experiments are detailed, which were made upon dogs, and in which the femoral and carotid arteries were transfixed by needles. Two experiments were made upon horses, but, immediately after the introduction of the needles, Mr. Phillips was obliged to leave London, so that he had no opportunity of examining the animals after they were destroyed for the purpose of ascertaining the results of the experiments in them. From these experiments Mr. Phillips feels himself justified in adducing the following conclusions:

"1. That acute inflammation is not easily excited in the tunics of arteries, even by the introduction of a foreign body.

"2. That obliteration of an artery may take place by the formation of a clot, without being preceded by inflammation of the internal tunic.

"3. That, when a clot is formed, and in apposition with the internal tunic of an artery, an adhesive inflammation is commenced; a vascular connexion succeeds; the clot becomes organised, and the obliteration of the artery is completed.

"4. That the introduction of a foreign body into an artery may occasion the formation of a clot or coagulum, from the blood circulating in that artery.

"5. That arteries may be obliterated with safety and certainty by the introduction into them of a foreign body.

"6. That, from the facility of introducing a common cylindrical needle, and the little irritation occasioned by its presence, such a body should have the preference.

"7. That, as the object is as much to interrupt the course of the circulating fluid as to excite a primary inflammation, two or more needles should be introduced.

"8. That, in the carotid and femoral arteries of dogs, if the needle be removed before the expiration of forty-eight hours, we shall not succeed in obliterating the artery; and that it is more prudent that they should remain sixty hours.

"9. That the presence of needles in four arteries, at the same moment, occasions a very trifling degree of general irritation.

"10. That perfect rest should be observed, at least during the presence of the needles in the arteries, or they may occasion suppuration.

"11. That an artery is not generally obliterated beyond the clot, and that the clot does not necessarily extend as far as the situation whence the first branch is given off.

"12. That care should be taken to remove the needles whenever any tendency to suppuration is manifested; and if obliteration be not effected, they may be again introduced."

Mr. Phillips was not aware, at the time he made these experiments, that Velpeau and Amussat had anticipated him to a certain extent. These gentlemen had employed large pins, by which excessive inflammation was produced; and, although in one or two cases coagula had been formed, these experiments had been deemed inconclusive.

In a short Appendix, Mr. Phillips states that, in the course of the experiments previously described, he became strongly impressed with the belief that arteries might be obliterated by galvanic action. Having satisfied himself of its power of exciting inflammation in any tissue, he conceived that, by the introduction of needles, for the purpose of conducting the galvanic fluid upon a given point, he might excite inflammation in that point; and he states, that the "experiments to which he has resorted have amply confirmed the justice of his anticipation."

Mr. Phillips has only made two experiments on the effect of galvanism upon the arteries and the circulating fluids. In the first, the femoral artery of a dog was laid bare just below Poupart's ligament, and two needles were placed in it: to one of these needles was attached the copper, to the other the zinc pole of a galvanic bartery. The contact was renewed three several times, at intervals of five minutes. In four hours and a half after the operation, the dog was destroyed: a small coagulum was found formed around the needles. This coagulum, which was not sufficiently large to be firmly applied to the internal parietes of the artery, was slightly adherent to the sides. Upon the internal tunic was found a stratum of coagulable lymph, and, when this was removed, the arterial parietes presented a considerable redness.—In the second experiment, the femoral artery was perforated by two needles, and the galvanic fluid applied as before. The dog was killed in twenty hours after the experiment, and in four hours after death the artery was examined, when it was found that a coagulum existed, of an inch in length, of considerable consistency, strongly adherent to the arterial parietes by means of a thick stratum of lymph; the internal tunic presented a vivid redness, of an arborescent character; the external was much inflamed.

In conclusion, Mr. Phillips expresses his hopes that experiments will be made by others, for the purpose of ascertaining whether equally important results will be obtained upon the human subject; and we agree with him that the extreme simplicity of the operation, and the total absence of danger in its application, place it entirely within the power of every practitioner. We regret that dogs were selected for the subjects of the experiments, as it is well known that the effects arising from wounding the arteries of these animals are totally different from those which would result in man. We have seen, for example, the femoral artery of a dog divided by a bistoury, without more than an ounce of blood being lost, and without any apparent inconvenience to the animal. Mr. Phillips will, no doubt, prosecute his very ingenious inquiries, and we beg to suggest that his experiments will be more satisfactory if they are made upon sheep or horses.

---

*Parts IV. and V. The Principles and Practice of Obstetric Medicine, in a Series of Systematic Dissertations on Midwifery, and on the Diseases of Women and Children. Illustrated by numerous Plates.* By David D. Davies, m.d., m.r.s.l., Professor of Midwifery in the University of London, &c.—John Taylor, London.

The fourth and fifth parts of this excellent practical work contain an instructive account of some of the diseases of the external genitals in the female; and there is one point the author refers to, which we have more than once mentioned, but its extreme importance forms sufficient apology for our again adverting to it. Female infants, and children of tender age, the offspring of parents totally free from syphilis, are occasionally subject to discharges from the vagina, very similar in their appearance, and attended by similar symptoms, to venereal gonorrhœa; and "it is moreover well known that children, similarly situated as to freedom even from a taint of syphilis, are not unfrequently the subjects of ulceration of their external genitals, which, under other circumstances, might be liable to great suspicion." It is of the utmost importance that practitioners should be acquainted with these facts; for it has not unfrequently happened that charges have been made against innocent persons, for the purpose of extorting money, of having attempted to violate the person of a young child, and the existence of a discharge from the vagina, or an ulceration from the child's pudendum, has been rashly taken as sufficient proof of the commission of the crime. Dr. Davies relates a case in point. During the early period of his life, a child, about eight years old, of low connexions and mendacious habits, was induced to prefer against a respectable minister of religion an accusation of an attempt to violate her person. Her friends averred that she had ulcerations of the pudendum in consequence of the imputed assault. The gentleman was imprisoned for many weeks: the grand jury ignored the bill, on the ground that the prisoner had proved himself totally free from the disease which he had been accused of communicating; and this conclusion was supported by other considerations, of a moral and circumstantial nature, which left no doubt of the innocence of the accused. Facts of a similar nature have been often recorded. The practitioner who is called upon to investigate such cases cannot but be sensible of the deep responsibility which attaches to him in the performance of his necessary, but disagreeable duty: he will be as anxious to detect the guilty as to protect the innocent from sordid conspiracy. The hints given by Dr. Davies will suggest the necessity of not hastily forming an opinion merely from any appearances that may be found on the person of the child. Children subject to such discharges and ulcerations are generally of a scrofulous constitution, and belonging to a class of society who have but little time to devote to their comfort or cleanliness.

The student will find some interesting remarks in the section on "certain troublesome Affections of the Urethra and its Orifice." The author relates a remarkable case of obstruction of the urethra, from a fungoid caruncolous growth which proceeded from the mucous membrane of the part.

A case occurred some years ago in our own practice, which we may here mention. An infant female was observed by its mother to suffer considerable pain during the act of making water. The external parts were carefully examined, but no appearance was detected which could explain the source of the child's sufferings. We were foiled in our attempts to relieve the child, from not being able to ascertain the real nature of the complaint. It happened, fortunately, that one day, during our visit, the child made water while we were examining the mucous membrane of the vagina, which had several white spots on its surface that had created anxiety in the minds of the friends. As usual, the little patient appeared to suffer pain in passing the urine, and, in the effort to expel the last drops, we perceived a small polypous growth projected from the orifice of the urethra. The moment the urine was entirely voided, the excrescence again passed into the urethra, and was completely hidden from sight. The case was troublesome, as it was only at the moment the child was making water that the excrescence could be seen. After many ineffectual efforts, a fine ligature was placed round it, and the child was well in a few days.

Another case of a similar kind occurs to our recollection, in which Mr. Stone was consulted; and here the same difficulty existed in seizing the small polypous growth, as it was only to be seen occasionally, and for a moment. This child was completely cured by Mr. S.: *we believe* he grasped the little excrescence with a pair of dressing forceps, and twisted it from its attachment to the urethra.

The subjects considered in the fifth part of the work, are "Pruritus of the external Genitals;" "the Situation, Structure, and Diseases of the Hymen and Carunculæ Myrtiformes."

We feel satisfied, from the parts already published of this work, that, when it is completed, it will be highly creditable to the talent and industry of the author, and very useful as a book of reference and study to those who possess it.

### COLLECTANEA.

